# MRAP2a Binds and Modulates Activity and Localisation of Prokineticin Receptor 1 in Zebrafish

**DOI:** 10.3390/ijms25147816

**Published:** 2024-07-17

**Authors:** Maria Rosaria Fullone, Daniela Maftei, Martina Vincenzi, Roberta Lattanzi, Rossella Miele

**Affiliations:** 1 Department of Biochemical Sciences “A. Rossi Fanelli”, Sapienza University of Rome, Piazzale Aldo Moro 5, 00185 Rome, Italy; mariarosaria.fullone@uniroma1.it; 2 Department of Physiology and Pharmacology “Vittorio Erspamer”, Sapienza University of Rome, Piazzale Aldo Moro 5, 00185 Rome, Italy; dani3la_maftei@yahoo.com (D.M.); martina.vincenzi@uniroma1.it (M.V.)

**Keywords:** prokineticin system, zebrafish PK2, MRAP2

## Abstract

The prokineticin system plays a role in hypothalamic neurons in the control of energy homeostasis. Prokineticin receptors (PKR1 and PKR2), like other G-protein-coupled receptors (GPCRs) are involved in the regulation of energy intake and expenditure and are modulated by the accessory membrane protein 2 of the melanocortin receptor (MRAP2). The aim of this work is to characterise the interaction and regulation of the non-melanocortin receptor PKR1 by MRAP2a in zebrafish (zMRAP2a) in order to use zebrafish as a model for the development of drugs targeting accessory proteins that can alter the localisation and activity of GPCRs. To this end, we first showed that zebrafish PKR1 (zPKR1) is able to interact with both zMRAP2a and human MRAP2 (hMRAP2). This interaction occurs between the N-terminal region of zPKR1 and the C-terminal domain of zMRAP2a, which shows high sequence identity with hMRAP2 and a similar propensity for dimer formation. Moreover, we demonstrated that in Chinese hamster ovary (CHO) cells, zMRAP2a or hMRAP2 are able to modulate zPKR1 activation induced by zebrafish PK2 (zPK2) resulting in an impaired ERK and STAT3 activation.

## 1. Introduction

Prokineticin receptors (PKRs, namely PKR1 and PKR2) are G protein-coupled receptors (GPCRs) that specifically bind prokineticin 1 (PK1) and prokineticin 2 (PK2) ligands. PKRs are able, by coupling all subtypes of G proteins such as Gαq/11, Gαs and Gαi, to mediate various signalling pathways, including protein kinase C, mitogen-activated protein kinase (ERK), phosphoinositide 3-kinase, STAT3 and protein kinase B [1,2,3,4,5,6,7,8,9]. In addition, PKRs interact constitutively with β-arrestin-2, although this interaction does not trigger internalisation of the receptor [10,11,12]. The complexity resulting from the presence of different isoforms of ligands and receptors [13,14,15,16] and from the ability of the receptors to couple different G proteins [1,2,3], dimerise [1,4] and interact with accessory proteins [1,4] allows the prokineticin system to perform specific functions in different tissues. The physiological functions of prokineticins include neurogenesis, angiogenesis, pain perception, and circadian rhythm regulation [17,18,19,20,21]. PK2 has also been shown to act as an adipokine. Initially, it was demonstrated that PK2 binding to PKR1 reduces food intake, stimulating the release of α-MSH and adipose tissue proliferation [22,23,24,25,26,27,28,29]. Recently, evidence has shown that inhibition of food intake by PK2 occurs through activation of amygdala PKR2 neurons [30].

The activation and localisation of PKRs is modulated by the accessory melanocortin receptor protein 2 (MRAP2), a single transmembrane protein that regulates several GPCRs involved in energy homeostasis [31,32,33,34,35,36]. The inhibitory effect of MRAP2 on the PKRs has been demonstrated both in vitro and in vivo, highlighting that the anorexigenic effect of PK2 is enhanced in *mrap2*-deficient mice characterised by severe obesity [37,38]. There are three different MRAPs in zebrafish: zMRAP, which is related to the tetrapod MRAP1, and zMRAP2a and zMRAP2b, which are classified as paralogues of mammalian MRAP2 [39,40,41,42,43,44,45]. Similarly, the prokineticin system has been identified in the zebrafish genome: two receptors corresponding to mammalian PKR1 and PKR2, named zPKR1 and zPKR2, respectively, and two ligands corresponding to mammalian PK1 and PK2, named zPK1 and zPK2 [46,47,48]. The aim of this study is to investigate the ability of zMRAP2a to interact with zPKR1 and to modulate its activity in order to use zebrafish as a model system for the study of diseases caused by dysregulation of the prokineticin system. Due to their rapid development and reproductive rate, zebrafish offer a unique opportunity for high-throughput drug screening to discover new compounds for the treatment of human diseases.

## 2. Results

### 2.1. Analysis of the mrap2a and mrap2b Genes

**Figure 1:**
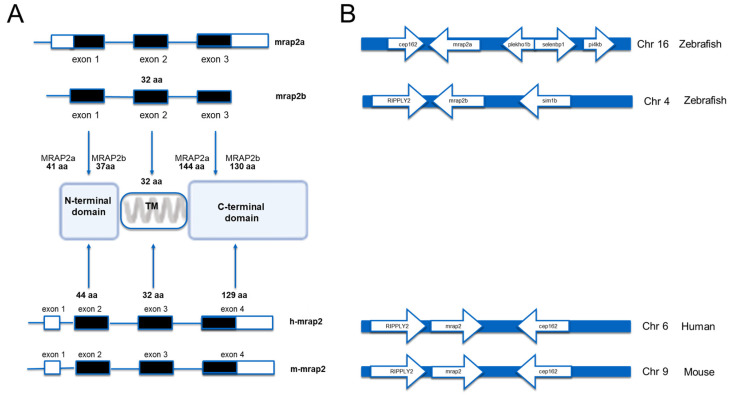
*mrap2a* and *mrap2b* genes of the zebrafish. (**A**) Schematic representation of zebrafish *mrap2a* and *mrap2b* genes and human *mrap2* and mouse *mrap2* genes. The coding exons are shown in black bars and the untranslated sequences are shown in white bars (**B**). Synteny analysis of human *mrap2* and mouse *mrap2* and *mrap2a* and *mrap2b* genes in zebrafish. Locus data are from the Ensembl database. RIPPLY2, ripply transcriptional repressor 2 (human, gene ID: 134701; mouse, gene ID: 382089; zebrafish, gene ID: 654447). *cep162*, centrosomal protein 162 (human; mouse; zebrafish, gene ID: 562035). *sim1b*, SIM bHLH transcription factor 1b (zebrafish, gene ID: 566656), *selenbp1*, selenium-binding protein 1 (zebrafish, gene ID: 393542); *plekho1b*, pleckstrin homology domain containing, family O member 1b (zebrafish, gene ID: 562940). *pi4kb*, phosphatidylinositol 4-kinase, catalytic, beta (zebrafish, Gene ID: 563201).

Zebrafish MRAP2a and MRAP2b, similarly to mammalian MRAP2s, have three distinct domains, each encoded by one exon. The three domains are a tyrosine-rich N-terminal region containing the conserved YEYY motif, a putative hydrophobic transmembrane domain of 23 amino acids (residues 34–56), and a C-terminal domain (Figure 1A).

The zebrafish *mrap2a* gene consists of three exons and two introns. The first exon contains a 5′ UTR (untranslated region) sequence and a sequence encoding the N-terminal region, which spans the first 41 amino acids; the second exon encodes the transmembrane region, which extends from the amino acid at position 42 to the amino acid at position 73; and the third exon contains a region encoding the C-terminal domain, which consists of 144 amino acids and the 3′ UTR sequence (Figure 1A). The zebrafish *mrap2b* gene also consists of three exons and two introns. As in *mrap2a*, the three exons encode the three N-terminal, the transmembrane and the C-terminal domains, but unlike the *mrap2a* exons, they do not contain the 5′ regions and the 3′ UTR. A synteny analysis was performed between the zebrafish *mrap2a* and *mrap2b* genes and the human *mrap2* and mouse *mrap2* genes. In humans and mice, the genes close to the *mrap2* gene are RIPPLY2 and *cep162*. Interestingly, the situation in zebrafish is in between: *mrap2a* is located next to *cep162* on chromosome 16 and *mrap2b* is next to RIPPLY2 on chromosome 4 (Figure 1B).

### 2.2. Analysis of the Physical Interaction between zPKR1 and zMRAP2a

**Figure 2:**
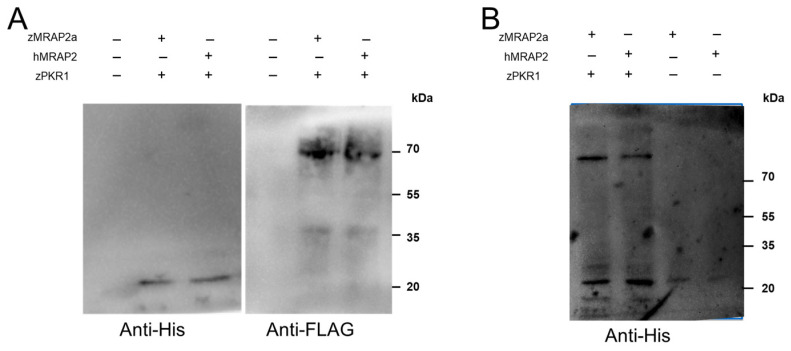
Interaction of zMRAP2a and hMRAP2 isoforms with zPKR1. (**A**) Detection of the interaction of zMRAP2a with the zPKR1 by immunoprecipitation. Membrane proteins immunoprecipitated with FLAG binding resin were resolved by SDS-PAGE. Immunoblots were probed with anti-His and anti-FLAG antibodies. (**B**) Crosslinking of zPKR1 with hMRAP2 and zMRAP2a. CHO cells expressing zPKR1 and zMRAP2a or hMRAP2 were incubated with dithiobis (succinimidyl propionate).

To determine whether zPKR1 and the accessory protein zMRAP2a interact directly, we performed co-precipitation experiments in which we co-expressed His-tagged zMRAP2 (zMRAP2a-His) and FLAG-tagged zPKR1 (zPKR1-FLAG) in CHO cells. zPKR1 was co-precipitated, subjected to SDS-PAGE analysis and transferred to a membrane. Blotting was then immunodetected with a commercial polyclonal antibody against a His-tag. Interestingly, zMRAP2a is able to co-precipitate with zPKR1, as shown in Figure 2A. The same experiment was repeated by co-expressing zPKR1 (zPKR1-FLAG) and hMRAP2 (hMRAP2-His) in Chinese hamster ovary (CHO) cells. Again, the ability of the zPKR1 to co-precipitate with hMRAP2 is evident (Figure 2A). To confirm the interaction between zPKR1 and zMRAP2a and to emphasise the ability of zPKR1 to also interact with hMRAP2, crosslinking experiments were performed. Membrane proteins from CHO cells co-expressing zPKR1/zMRAP2a and zPKR1/hMRAP2 were exposed to the crosslinker dithiobis (succinimidyl propionate). The proteins were separated by SDS-PAGE, transferred to the membrane by blotting and then tested with anti-His antibodies (Figure 2B). In the lanes corresponding to the membrane protein of CHO cells expressing zPKR1/zMRAP2a, there is a band with a size comparable to the molecular weight calculated for the crosslinked zPKR1/zMRAP2a complex. In the lane corresponding to membrane proteins extracted from CHO cells expressing zPKR1/hMRAP2, a band is also seen, but with much lower intensity. The results were confirmed by three independent replicates of the experiments.

### 2.3. Expression of the C-Terminal Region of Zebrafish MRAP2a in E. coli

Previous studies have allowed us to identify the region of hMRAP2 that can mediate the interaction with hPKR2 [34,35].

**Figure 3:**
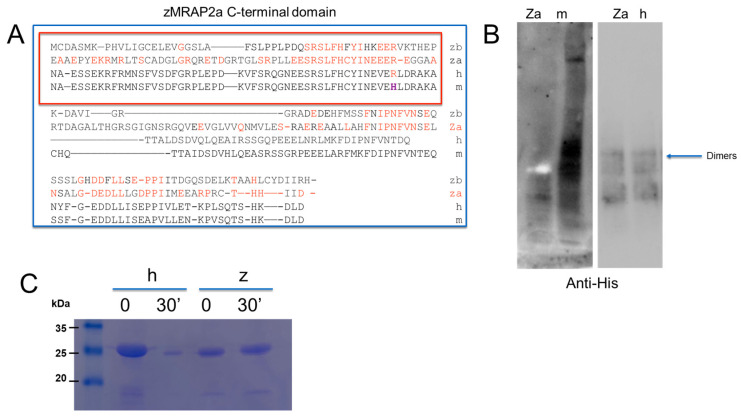
Biochemical analysis of the zCT-MRAP2a domain. (**A**) Alignments of the C-terminal domain of human (h), mouse (m) and zebrafish (za, zb) MRAP2. The alignment of the region important for the interaction of MRAP2 with PKRs is shown in the red box. In red the amino acids that are conserved in zebrafish and mouse with respect to hPKR1 and hPKR2. In purple indicates an amino acid that is present in mice and is important for the conformation of the protein [35]. (**B**) Blue native PAGE of the C-terminal domains of human (h), mouse (m), and zebrafish (za) MRAP2 expressed in *E. coli*. (**C**) SDS-PAGE analysis of hCT-MRAP2 and zCT-MRAP2, limited time course of proteolysis.

Comparison of the amino acid sequences of the C-terminal (CT) domain of zMRAP2a with zMRAP2b, hMRAP2 and mMRAP2 shows that this region involved in PKR binding is extremely conserved in zMRAP2a, but not in zMRAP2b (Figure 3A).

To test whether the CT domain of zMRAP2a is important for the interaction with zPKR1, a deletion mutant of zMRAP2 (zCT-MRAP2a) was generated containing the region from residue 73 to residue 217 encoded by the third exon. The protein was expressed in *E. coli*. Electrophoretic analysis showed that the purified zCT-MRAP2a protein has a molecular mass of 10 kDa, close to the values calculated on the basis of the genetic sequence, and that it retains the ability to dimerise. Indeed, electrophoretic analysis shows that the protein migrates as a dimer even under denaturing conditions, as previously described for the CT domain of the hMRAP2 protein [34,35]. The ability of the zCT-MRAP2 protein to form higher-order oligomers was also emphasised by the blue native PAGE. In this way, it was possible to recognise the presence of structures that are most likely to be dimers, although the prediction of molecular weights is only approximate (Figure 3B). To compare the conformation of the CT domain of zMRAP2a with human CT domains (hCT-MRAP2), we performed limited proteolysis. The results, which show different effects of proteolysis, indicate differences in the conformation of the two proteins (Figure 3C).

### 2.4. Analysis of the Role of the N-Terminal Region of hPKR1 and zPKR1 for the zMRAP2a Interaction

**Figure 4:**
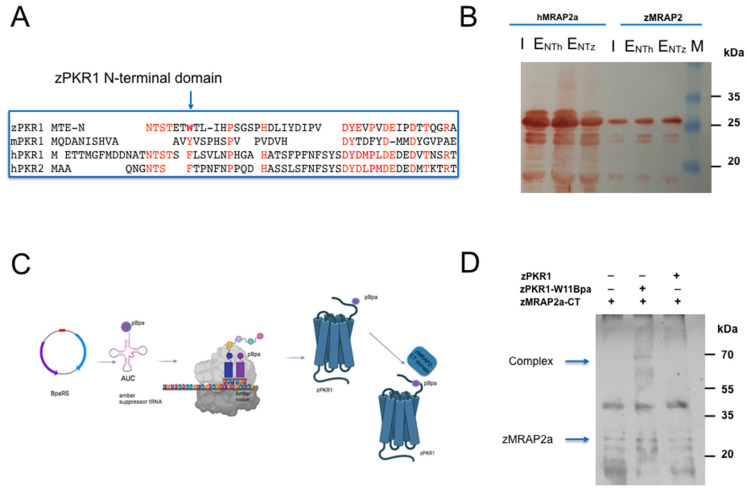
Role of N-Terminal region of zPKR1 for zMRAP2a and hMRAP2 interaction. (**A**) Alignments of the hPKR1, hPKR2, mPKR1 and zPKR1 N-terminal sequence. In red the amino acids that are conserved in zebrafish and mouse with respect to hPKR1 and hPKR2, in bold the amino acid that was replaced by photoreactive p-benzoyl-L-phenilalanine. (**B**) GST pull-down experiments. The zPKR1-NT-GST and hPKR1-NT-GST were used to pull down CT-domains of zebrafish zMRAP2a and of hMRAP2. The eluate obtained using zPKR1-NT-GST (E_NTz_) and eluate obtained using hPKR1-NT-GST(E_NTh_) were analysed by Western blotting analysis with anti-His antibody. The negative control (E_GST_) was obtained using GST to pull down zMRAP2a. (**C**) Schematic representation of the amber codon suppression technology for genetic introduction of the photoreactive p-benzoyl-L-phenilalanine (pBpa) directly into expressed zPKR1 in a yeast cell system. (**D**) Crosslinking of zPKR1-WT and the zPKR1-W11Bpa with zCT-MRAP2a domain. Membranes prepared from *P. pastoris* cells expressing the zPKR1-W11Bpa and zPKR1-WT were incubated with zCT-MRAP2a domain. The membrane proteins were immunoblotted and analysed with anti-His antibodies.

The N-terminal (NT) region of hPKR1 is involved in hMRAP2 interaction [34,35]. We analysed the interaction between the zCT-MRAP2a domain and the NT region of zPKR1 using glutathione S-transferase (GST) pull-down experiments (Figure 4A). The zPKR1-NT-GST fusion protein was purified on glutathione agarose and then used in direct binding experiments with the His-tagged zCT-MRAP2a domain. The zPKR1-NT-GST fusion protein is able to pull down zCT-MRAP2a, a deletion mutant containing only the CT region of zMRAP2a, as shown in Figure 4A, demonstrating their interaction. In contrast, GST alone is unable to knock down zCT-MRAP2 (Figure 4B). The experiment was repeated with the fusion protein zNT-PKR1-GST and hCT-MRAP2. The results show that the zPKR1-NT-GST fusion protein is also capable of pulling down hCT-MRAP2. We then analysed the role of the aromatic tryptophan residue in position 11 of the amino-terminal region of zPKR1 in binding zMRAP2. The presence of the aromatic residue in the N-terminus of PKR1 is extremely conserved from an evolutionary point of view (Figure 4A). In a yeast cell-based system, we used codon suppression technology to introduce photoreactive p-benzoyl-L-phenylalanine (Bpa) at position 11 directly into the expressed zPKR1 protein (Figure 4C). The plasmid encoding the FLAG-tagged amber zPKR1-W11 mutant was transformed in *P. pastoris* with the pREAV-P FLD1-pBpaRS plasmid encoding the orthogonal amber suppressor tRNA synthetase/tRNA pair, which was genetically modified to allow the incorporation of Bpa [49,50,51] (Figure 4C). Next, the membranes of *P. pastoris* cells expressing the zPKR1-WT and zPKR1-W11Bpa were incubated with or without the zCT-MRAP2a domain and then irradiated with UV light. The membranes were fractionated by SDS-PAGE, blotted, and then tested with an antibody against the His-tag to detect the binding of ligands to the Bpa-labelled receptor. The results show that a distinct band of the size expected for the zCT-MRAP2a-zPKR1 complex was only detected in the presence of zPKR1-W11Bpa (Figure 4D). These results were confirmed by three independent replicates of this experiment.

### 2.5. Modulation of zPKR1a and hPKR1 Signalling by zMRAP2a and hMRAP2

**Figure 5:**
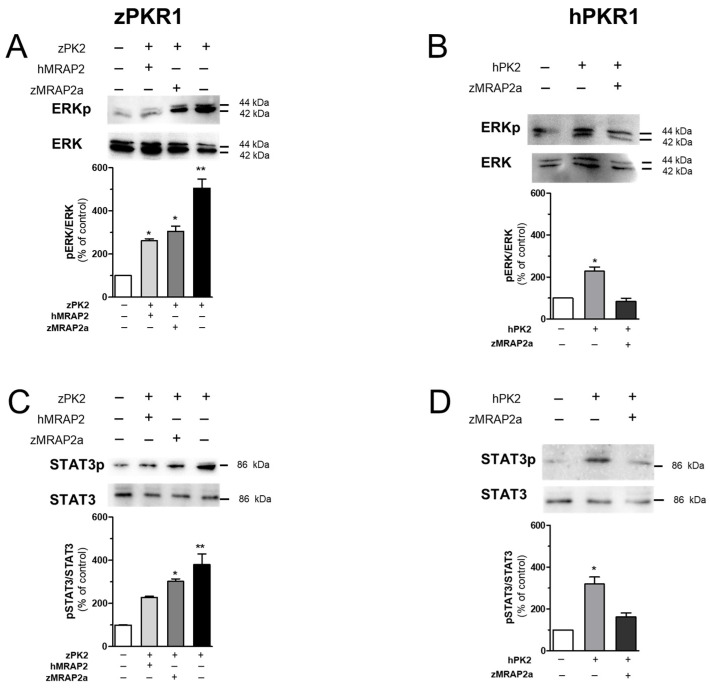
Analysis of ERK and STAT3 activation in CHO cells. (**A**,**B**) Analysis of ERK1/2 phosphorylation in CHO cells transfected with zPKR1 or hPKR1. Densitometric plots show pERK1/2 and ERK1/2 protein levels 10 min after treatment with zPK2 or hPK2 (100 nM). The bar graphs show the pERK1/2/ERK1/2 ratio and the percentage increase compared to unstimulated cells (CTRL). (**C**,**D**) Densitometric plots show phospho-STAT3 (pSTAT3) and STAT3 protein levels in CHO cells expressing zPKR1 or hPKR1 after 1 h of treatment with zPK2 or hPK2 (100 nM). Data are presented as the ratio of pSTAT3 to total STAT3 protein and plotted as a percentage increase compared to CTRL. The bars show the mean values ± SEM of the three experimental conditions. For statistical analysis, a one-way ANOVA followed by Tukey’s test for multiple comparisons was used. * *p* < 0.05, ** *p* < 0.01 versus CTRL.

It is known that hPK2 in CHO cells stably transfected with hPKR1 or hPKR2 is able to activate ERK and STAT3 signalling pathways via Gαi coupling [1,2,3,8,9]. We have recently shown that treatment with zPK2 can also induce activation of ERK and STAT3 through the expression of zPKR1 in CHO [44]. To determine whether the interaction of zPKR1 with zMRAP2a has pharmacological significance, we analysed ERK and STAT3 phosphorylation by zPK2 stimulation of CHO cells transiently expressing zPKR1. In the absence of zMRPA2a, it is possible to highlight that zPK2 induces phosphorylation of ERK and STAT3 upon binding of zPKR1. However, co-transfection of MRAP2a strongly reduces the effect of zPK2 on the activation of zPKR1 (Figure 5A,C). We also co-expressed the zPKR1with hMRAP2 in CHO cells and stimulated the cells with zPK2. Co-expression of zPKR1 and hMRAP2 inhibited ERK and STAT3 activation, with an effect that was much stronger than that induced by zPKR1 and zMRAP2 co-expression. Similarly, in zebrafish, co-expression of hPKR1 and zMRAP2a inhibited ERK and STAT3 activation after hPK2 incubation with a weak effect (Figure 5B,D).

### 2.6. Modulation of hPKR1 Localization by zMRAP2a and hMRAP2

**Figure 6:**
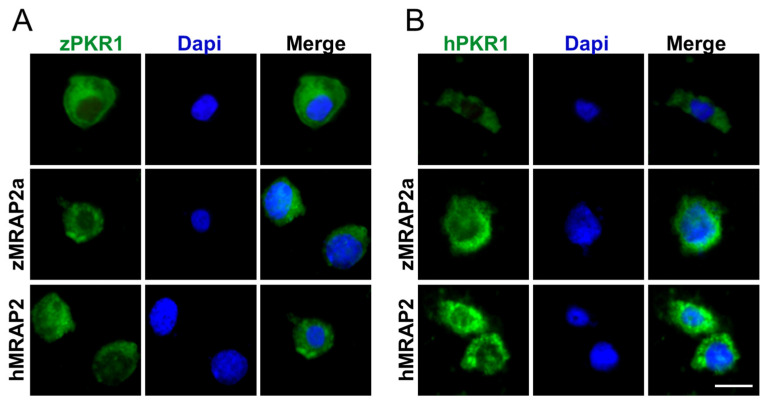
(**A**) Representative immunofluorescence images of CHO cells transfected with zPKR1-GFP (green) and zMRAP2a or hMRAP2. (**B**) Representative immunofluorescence images of CHO cells transfected with hPKR1-GFP (green) and zMRAP2a or hMRAP2. Scale bar 10 μm. The cell nuclei were counterstained with DAPI (blue).

Analysis of immunofluorescence experiments shows that, in the absence of zMRAP2a, zPKR1 is pre-dominantly localised in the plasmatic membrane. The co-expression of zPKR1 with zMRAP2a or hMRAP2 promotes the accumulation of zPKR1 inside the cells even if the effect is more evident in the presence of zMRAP2a (Figure 6A). The co-expression of hPKR1 and hMRAP2 promotes the accumulation of hPKR1 inside the cells but the effect is not that pronounced in the presence of zMRAP2a (Figure 6B).

## 3. Discussion

Genes of the prokineticin system have been identified in the zebrafish genome, in particular two protein-coding genes *pkr1a* and *pkr1b* (zPKR1 and zPKR2), corresponding to the mammalian receptors PKR1 and PKR2, and two protein-coding genes (zPK1 and zPK2), corresponding to the mammalian ligands PK1 and PK2 [46,47,48]. zPKR1 has been shown to mediate PK2-induced angiogenesis, and its expression, like that of zPK2, is regulated by a transcription factor called TBX20 [52]. In contrast, zPKR2 plays a crucial role in the migration of GnRH neurons and the ontogenesis of GnRH in zebrafish and mammals. Indeed, ablation of the *pkr1b* gene leads to a reduction in the dorsal projections of GnRH neurons; although, unlike in mammals, this does not cause structural changes in the gonads [53,54,55,56].

The MRAP2, a small transmembrane protein widely distributed in numerous tissues, contributes to the regulation of central neuronal appetite and peripheral energy homeostasis, by regulating the physiological actions of melanocortin receptors and many other GPCRs such as PKR1, orexin receptor and ghrelin receptor (GHSR1a) [40]. As in other teleost species, two MRAP2 paralogues have been characterised in zebrafish [43,44,45,57], namely zMRAP2a and zMRAP2b, whereas in other lower vertebrates, including tilapia, sea lamprey and grouper, only one paralogue is present, as in mammals [58,59,60,61,62]. Therefore, the gene encoding MRAP2, like other genes in the zebrafish genome, has undergone genetic duplication, suggesting a more complex phylogenetic process than in mammals. We were able to show that the synthesis of zMRAP2a and zMRAP2b is conserved in zebrafish compared to MRAP2 in humans and mice. This result allowed us to give a strong indication of the orthology between the two zebrafish genes *mrap2a* and *mrap2b* and the mammalian *mrap2* genes.

MRAP2 has different effects on melanocortin receptors in different species [58,59,60,61,62]. Therefore, it is of interest to determine the effect of MRAP2 on zPKR1 and compare it with the well-characterised effect in mammals. Using crosslinking and GST pull-down experiments, we were able to demonstrate that zMRAP2a interacts with zPKR1. Similarly, zPKR1 is able to interact with hMRAP2.

We then expressed the C-terminal domain of zMRAP2a, which has been shown to be important in modulating zebrafish melanocortin receptor 4 (zMC4R) activity [45], by replacing the CT of zMRAP2a with that of zMRAP2b. MRAP2a and zMRAP2b modulate zMC4R signalling in opposite ways: zMRAP2a significantly inhibits zMC4R signalling, whereas zMRAP2b enhances it in a dose-dependent manner. The C-terminal domain of hMRAP2, which is highly conserved in evolution across species, plays an essential role in regulating both the binding and activity of human PKRs [12,34,35,36]. The data obtained show that this region is highly conserved in zMRAP2a, whereas it is only partially conserved in zMRAP2b, and that is able to mediate the interaction with the N-terminal region of zPKR1. We also emphasised that the CTregion of MRAP2a interacts with a specific residue of tryptophan in position 11 of PKR1 using codon suppression technology. We expressed and characterised the CT domain of zMRAP2a demonstrating that it adopts a different conformation and has a different propensity to dimerise with respect to human and mouse MRAP2. MRAP2a, as hMRAP2, is not only able to modulate the activity of the zPKR1, but it is also able to influence its sur-face expression.

## 4. Materials and Methods

### 4.1. Drugs and Reagents

Dulbecco’s Modified Eagle Medium with F-12 supplement (DMEM/F-12) media, foetal bovine serum (FBS), phosphatases inhibitor cocktails, penicillin/streptomycin, L-glutamine, poly-D-lysine, Trypsin Glutathione-Sepharose beads, crosslinker dithiobis (succinimidyl propionate), and N-cyclohexyl-3-aminopropanesulfonic acid were obtained from Sigma-Aldrich (Sigma, St. Louis, MO, USA). Lipofectamine 2000 (Lipo) was obtained from Invitrogen Life Technologies (Thermo Fisher Scientific, Monza, Italy). Enzymes used for molecular cloning and an enhanced chemiluminescence detection kit were obtained from Roche Molecular Biochemicals (Monza, Italy). DreamTaq DNA, BCA Protein Analysis Reagents, Native PAGE Novex 3% to 16% Bis–Tris gels and Trizol reagent were obtained from Thermo Fisher Scientific (Monza, Italy). Nitrocellulose membranes (0.45 μm; Hybond-C Extra) were obtained from Amersham Pharmacia Biotech Amersham, United Kingdom). The His-tagged protein purification kit was obtained from Novagen (Darmstadt, Germany).

### 4.2. Antibodies

The following primary antibodies were used in this study. From Sigma Aldrich: mouse monoclonal antibody anti-FLAG (F1804) and anti-polyHistidine–Peroxidase (A7058). From Invitrogen-Thermo Fisher Scientific (Monza, Italy): rabbit polyclonal antibody anti-ERK (#44-654), rabbit polyclonal antibody anti-pERK (#44-680), mouse anti-STAT3 (MA1-13,042), and rabbit anti-pSTAT3 (Tyr705) (#44-380G).

### 4.3. Expression Constructs

For production of the zCT-MRAP2 deletion mutant in *E. coli* we used pet 28zCT-MRAP2. This was obtained by cloning a fragment via PCR with oligonucleotides MRAP2a rev EcoRI and MRAP2a CT fw BamHI (Table 1) using plasmid XM_021473026.1 (Genscript, Piscatawey, NJ, USA) as a template in pet28 digested with BamHI and Eco-rI. For production of hCT-MRAP2 and mCT-MRAP2 deletion mutant in *E. coli* we used pet28 hCT-MRAP2 and pet28 mCT-MRAP2, as described in [32,33].

For cell culture experiments and to express zPKR1, NM_001173406.1 purchased from Genscript was used. To express MRAP2a, we used XM_021473026.1 plasmid (Genscript).

zPKR1 fused with GFP was obtained by cloning the cDNA amplified by PCR using zPKR1-up-BamHI and zPKR1-Dw-AgeI oligonucleotides (Table 1) in pEGFP-N1.

### 4.4. Sequence Homology Comparison and Synteny Analysis

Multiple alignments of protein sequences were generated by based on Clustal Omega program (https://www.ebi.ac.uk/jdispatcher/msa/clustalo, accessed on 16 April 2024) using default setting in preferences. The gene locus information of MRAP2a (gene ID: 100003330) and MRAP2b (gene ID: 101884805) genes in zebrafish, mouse MRAP2 (gene ID: 244958) and human MRAP2 (gene ID: 112609) were obtained from the Ensembl database (www.ensembl.org, accessed on 16 April 2024), respectively.

### 4.5. Co-Precipitation of Membrane Proteins

Solubilised membranes from cells co-expressing zPKR1 and MRAP2a or zPKR1 and hMRAP2 were co-precipitated using a FLAG-binding resin. The eluate was analysed using SDS-PAGE on a 12% gel and was transferred to a polyvinylidene difluoride membrane. The membrane was probed with a polyclonal anti-His antibody (1:5000) or a monoclonal anti-FLAG antibody (1:1000).

### 4.6. Crosslinking

CHO cells were transiently transfected with pcDNA zPKR1/pcDNA zMRAP2a or pcDNA zPKR1/pCMV hMRAP2. After transfection (24 h), the cells were incubated at room temperature with an amine-reactive crosslinker dithiobis (succinimidyl propionate) at a final concentration of 2 mM. Crosslinking was terminated by washing the cells twice with 50 mM Tris/HCl, pH 7.4. Finally, the cells were lysed in 0.5 mL 50 mM Hepes, pH 7.4, 50 mM NaCl, 10% (*v*/*v*) glycerol, 0.5% (*v*/*v*) Nonidet P40, 2 mM EDTA, 1 mM PMSF, 10 μg/mL benzamidine, 5 μg/mL soybean trypsin inhibitor, and 5 μg/mL leupeptin.

### 4.7. Expression of zCT-MRAP2a in E. coli

After growing *E. coli* cultures expressing the CT domains (human (hCT-MRAP2), human MRAP2 R125H mutant (hCT-R125H MRAP2) and zebrafish (zCT-MRAP2)) to an optical density of 0.5 at 600 nm, isopropyl-β-D-1-thiogalactopyranoside (IPTG) at a concentration of 0.1 mM was added. The cultures were then incubated for a further 4 h at 28 °C. After purification of the proteins, using a His-tagged protein purification kit, the recombinant proteins were analysed by SDS-PAGE and Western blotting with an anti-His antibody (1:5000).

### 4.8. Blue Native PAGE

A total of 20 μg of purified CT-MRAP2 domains were loaded onto Native PAGE Novex 3% to 16% Bis-Tris gels. After electrophoresis at 0 °C using the buffers and conditions specified by the manufacturer, the proteins were transferred to a polyvinylidene fluoride membrane. The membrane was destained in methanol for 3 min, rinsed in T-TBS pH 7.4 and then immunoblotted with monoclonal anti-His (1:5000).

### 4.9. Limited Proteolysis Experiments

Purified CT-MRAP2 domains (5 μg) were incubated with trypsin from bovine pancreas (0.002 μg/L) at 20 °C in 50 mM Hepes, pH 8.0, with 300 mM NaCl and 5% glycerol (*v*/*v*). The proteolytic reaction was stopped by the addition of SDS. After boiling, the samples were analysed by SDS-PAGE.

### 4.10. Glutathione S-Transferase (GST) Pull-Down

The zPKR1-GST protein was obtained by fusion of the 44 amino acids from the amino-terminal sequence of zPKR1 with GST as described in [48]. The PKR1-GST proteins were obtained by fusion of the 57 amino acids from the amino-terminal sequence of hPKR1 with GST [15].

The zPKR1-GST protein was purified from *E. coli* cell extracts by affinity chromatography using glutathione-Sepharose beads according to the manufacturer’s instructions. In brief, 30 μL of the glutathione-Sepharose bead suspension was equilibrated in buffer A (PBS, 1% Nonidet P 40, 1 mM EDTA supplemented with protease inhibitor mixture) for 1 h at 4 °C with constant stirring in the presence of the cell lysate. After determining the concentrations of GST fusion proteins, using the BCA method, the beads were incubated for 4 h with an equivalent amount of purified hPK2 or zPK2.

After washing the beads, the bound proteins were eluted with GSH according to the procedure indicated by GE Healthcare. The elution samples were analysed on 15% SDS-PAGE gels and by Western blotting using anti-His antibodies (1:5000).

### 4.11. Expression of zPKR1-W11Bpa Mutant in P. pastoris

For expression in *P. pastoris*, the cDNA encoding zPKR1 was inserted into pADE (zPKR1 pADE). To obtain the mutant zPKR1-W11Bpa, the cDNA was amplified using zPKR1-pcDNA as a template using the oligonucleotides zPKR1uaa-up-BamHI and zPKR1-Dw-XhoI. The PCR product was cloned into pADE (W11amber-zPKR1 pADE). The pREAV-P FLD1-pBpaRS plasmid linearised with AatII was transformed into *P. pastoris* JC300 by electroporation to allow integration into the ARG4 locus of the *P. pastoris* genome [49,50,51]. W11amber-zPKR1 pADE or zPKR1 pADE were linearised with EcoNI to facilitate integration into the ADE1 locus of the *P. pastoris* genome. Transformants were selected on selective minimal MD medium (1.34% YNB, 2 microgram/L biotin, 1% dextrose and 1.5% agar supplemented with His). Integration of the plasmids was confirmed by PCR on genomic DNA.

For expression of zPKR1 and zPKR1-W11Bpa proteins, yeasts were grown for 24 h at 30 °C in BMG medium (1% yeast extract, 2% peptone, 50 mM potassium phosphate buffer pH 6) in the presence of 1% glycerol and were then incubated for 120 h at 30 °C in the presence of 1% methanol and 2 mM Bpa.

### 4.12. Yeast Membrane Preparation and Crosslinking

Lysis of cells and extraction of membrane proteins were performed as described in [15]. Cells expressing zPKR1-W11Bpa or zPKR1 and grown in the presence or absence of Bpa (1 mM) were suspended in lysis buffer (50 mM MOPS, pH 7.5; 150 mM NaCl) and lysed by vortexing with glass beads. Unlysed cells were removed by centrifugation at 700× *g*, and the membrane fraction was collected by centrifugation at 150,000× *g* for 40 min. The membranes were incubated with zCT-MRAP2a for 30 min at room temperature. After inducing crosslinking by irradiating the membranes with UV light at 365 nm using a Stratalinker (Stratagene, La Jolla, CA, USA) for three 15 min intervals, they were washed three times with CAPS buffer (10 mM N-cyclohexyl-3-aminopropanesulfonic acid at pH 10). The resulting complexes were precipitated by centrifugation and the pellet was resuspended in lysis buffer in the presence of 1% Triton X-100. After one hour of incubation at room temperature, the solution was centrifuged again at 150,000× *g* and the supernatant was analyzed by Western blot with anti-His antibodies.

### 4.13. zPK2 Production in P. pastoris

zPK2 was expressed as a His-tagged protein in *P. pastoris* as described in [48]. Crude culture supernatants were purified using a kit for His-tagged protein. Proteins were eluted with 20 mM Tris-HCl (pH 7.0), 300 mM NaCl, 0.25 M imidazole. After dialysis against 20 mM Tris-HCl (pH 7.0) buffer, recombinant protein was analysed using Tris-Tricine SDS-PAGE gel and by Western blot.

### 4.14. CHO Cell Culture and Stimulation

CHO cells were cultured at a density of or 4 × 10^4^ per well in 24-well plates on poly-D-lysine coated coverslips and 2 × 10^5^ per well in 6-well plates and allowed to grow until they reached 70–80% confluence. Cells were grown in Dulbecco Modified Eagle Medium/Nutrient mixture F-12 Ham containing 100 U/mL penicillin/streptomycin, 10% foetal bovine serum (FBS), and 2 mM L-glutamine at 37 °C and 5% CO_2_. Transient transfection with the plasmids hMRAP2 pCMV and zMRAP2a, hPKR1-GFP, zPKR1-GFP pcDNA was performed using Lipofectamine 2000 according to the manufacturer’s instructions. In brief, cells were incubated with the Lipo-DNA complex for 24 h at 37 °C and 5% CO_2_. A total of 24 h after transfection, cells were deprived of serum and stimulated with zPK2 (100 nM) and hPK2 (100 nM) for 10 min and 1 h at 37 °C, 5% CO_2_.

#### 4.14.1. Analysis of STAT3 and ERK Activation

The cells were then lysed, and total protein was quantified by the Bradford method. Proteins were separated by electrophoresis and then transferred to a nitrocellulose membrane (TCM) and blocked in 1% non-fat milk and 1% BSA/Tris-buffered saline containing 0.10% Tween-20 (TBS-T pH 7.4) for 1 h at room temperature. Subsequently, the membranes were incubated overnight at 4 °C with the appropriate primary antibodies diluted to a ratio of 1:1000 in the blocking solution. Membranes were then incubated with an anti-mouse or anti-rabbit IgG HRP-linked secondary antibody. The immunoreactive signals were visualised using an enhanced chemiluminescence system.

#### 4.14.2. Immunofluorescence

CHO cells were fixed in PFA 4%, washed in 1X phosphate buffered solution (PBS) and stained for cell nuclei with DAPI (Sigma-Aldrich). The GFP fluorescence signal was analysed using an Eclipse E600 fluorescence microscope (Nikon Instruments, Tokio, Japan) connected to a QImaging camera with 64-bit NIS-Elements BR 3.2 software.

### 4.15. Data Analysis

The data were plotted and analysed using GraphPad Prism 7 for Windows. All results were expressed as mean ± SEM. Statistical analyses were performed using one-way ANOVA followed by Tukey’s multiple comparisons post-test. Differences were considered significant at *p* < 0.001.

## 5. Conclusions

MRAP2, originally isolated as a melanocorticoid receptor-regulating protein, may act as a functional link in hypothalamic neurons to control energy homeostasis by synergistically modulating a large group of GPCRs. In this work, for the first time, we characterised the interaction and regulation of a non-melanocortin receptor, zPKR1, by zMRAP2a in zebrafish. This system could be used for the development of drugs targeting GPCR accessory proteins for clinical treatments [63,64,65].

## Figures and Tables

**Table 1:** Oligonucleotides used in this study.

Oligonucleotides	Sequences
zPKR1-up-BamHI	5′-AAGGATCCATGACTGAAAAACAACACAAGC-3′
PKR1-Dw-XhoI	5′-TTGAATTCGGCCACGAAAAACGCTCGTCCC-3′
MRAP2a rev EcoRI	5′-TTAGAATTCATCAATGATATGATGAGTACAGC-3′
MRAP2a CT fw BamHI	5′-GACCATGGATGGCAGCCCAGAATGG-3′

## Data Availability

Data will be made available on request by corresponding authors.

## References

[1] Vincenzi M., Kremić A., Jouve A., Lattanzi R., Miele R., Benharouga M., Alfaidy N., Migrenne-Li S., Kanthasamy A.G., Porcionatto M. (2023). Therapeutic Potential of Targeting Prokineticin Receptors in Diseases. Pharmacol. Rev..

[2] Monnier J., Samson M. (2008). Cytokine properties of prokineticins. FEBS J..

[3] Negri L., Ferrara N. (2018). The Prokineticins: Neuromodulators and Mediators of Inflammation and Myeloid Cell-Dependent Angiogenesis. Physiol. Rev..

[4] Lattanzi R., Miele R. (2022). Prokineticin-Receptor Network: Mechanisms of Regulation. Life.

[5] Cook I.H., Evans J., Maldonado-Perez D., Critchley H.O., Sales K.J., Jabbour H.N. (2010). Prokineticin-1 (PROK1) modulates interleukin (IL)-11 expression via prokineticin receptor 1 (PROKR1) and the calcineurin/NFAT signalling pathway. Mol. Hum. Reprod..

[6] Zhong C., Qu X., Tan M., Meng Y.G., Ferrara N. (2009). Characterization and regulation of Bv8 in human blood cells. Clin. Cancer Res..

[7] Lattanzi R., Maftei D., Fullone M.R., Miele R. (2021). *Trypanosoma cruzi* trans-sialidase inducesSTAT3 and ERK activation by prokineticin receptor 2 binding. Cell Biochem. Funct..

[8] Xin H., Lu R., Lee H., Zhang W., Zhang C., Deng J., Liu Y., Shen S., Wagner K.U., Forman S. (2013). G-protein-coupled receptor agonist Bv8/prokineticin-2 and STAT3 protein form a feed-forward loop in both normal and malignant myeloid cells. J. Biol. Chem..

[9] Qu X., Zhuang G., Yu L., Meng G., Ferrara N. (2012). Induction of Bv8 expression by granulocyte colony-stimulating factor in CD11b+Gr1+ cells: Key role of Stat3 signaling. J. Biol. Chem..

[10] Yin W., Liu H., Peng Z., Chen D., Li J., Li J.-D. (2014). Mechanisms that underlie the internalization and extracellular signal regulated kinase 1/2 activation by PKR2 receptor. Cell. Signal..

[11] Casella I., Ambrosio C. (2021). Prokineticin receptors interact unselectively with several G protein sub-types but bind selectively to beta-arrestin 2. Cell. Signal..

[12] Lattanzi R., Casella I., Fullone M.R., Maftei D., Miele R. (2024). MRAP2 Inhibits β-Arrestin-2 Recruitment to the Prokineticin Receptor 2. Curr. Issues Mol. Biol..

[13] Chen J., Kuei C., Sutton S., Wilson S., Yu J., Kamme F., Mazur C., Lovenberg T., Liu C. (2005). Identification and pharmacological characterization of prokineticin 2 beta as a selective ligand for prokineticin receptor 1. Mol. Pharmacol..

[14] Lattanzi R., Maftei D., Negri L., Fusco I., Miele R. (2018). PK2β ligand, a splice variant of prokineticin 2, is able to modulate and drive signaling through PKR1 receptor. Neuropeptides.

[15] Lattanzi R., Maftei D., Vincenzi M., Fullone M.R., Miele R. (2022). Identification and Characterization of a New Splicing Variant of Prokineticin 2. Life.

[16] Lattanzi R., Maftei D., Fullone M.R., Miele R. (2019). Identification and characterization of Prokineticin receptor 2 splicing TM4-7 variant and its modulation in an animal model of Alzheimer’s disease. Neuropeptides.

[17] Zhou Q.-Y., Cheng M.Y. (2005). Prokineticin 2 and circadian clock output. Prokineticin 2 and circadian clock output. FEBS J..

[18] Goryszewska E., Kaczynski P., Balboni G., Waclawik A. (2020). Prokineticin 1–prokineticin receptor 1 signaling promotes angiogenesis in the porcine endometrium during pregnancy. Biol. Reprod..

[19] Lattanzi R., Severini C., Miele R. (2022). Prokineticin 2 in cancer-related inflammation. Cancer Lett..

[20] Zhao Y., Wu J., Wang X., Jia H., Chen D.N., Li J.D. (2019). Prokineticins and their G protein-coupled receptors in health and disease. Prog. Mol. Biol. Transl. Sci..

[21] Lattanzi R., Miele R. (2022). Non-Peptide Agonists and Antagonists of the Prokineticin Receptors. Curr. Issues Mol. Biol..

[22] Gardiner J.V., Bataveljic A., Patel N.A., Bewick G.A., Roy D., Campbell D., Greenwood H.C., Murphy K.G., Hameed S., Jethwa P.H. (2010). Prokineticin 2 is a hypothalamic neuropeptide that potently inhibits food intake. Diabetes.

[23] Sohn J.W. (2015). Network of hypothalamic neurons that control appetite. BMB Rep..

[24] Von Hunolstein J.J., Nebigil C.G. (2015). Can prokineticin prevent obesity and insulin resistance?. Curr. Opin. Endocrinol. Diabetes Obes..

[25] Wang H., Jia Y., Yu X., Peng L., Mou C., Song Z., Chen D., Li X. (2021). Circulating Prokineticin 2 Levels Are Increased in Children with Obesity and Correlated with Insulin Resistance. Int. J. Endocrinol..

[26] Zhou W., Li J.-D., Hu W.-P., Cheng M.Y., Zhou Q.-Y. (2012). Prokineticin 2 is involved in the thermoregulation and energy expenditure. Regul. Pept..

[27] Wang Y., Guo X., Ma H., Lu L., Zhang R. (2016). Prokineticin-2 is associated with metabolic syndrome in a middle-aged and elderly Chinese population. Lipids Health Dis..

[28] Magnan C., Migrenne-Li S. (2021). Pleiotropic effects of prokineticin 2 in the control of energy metabolism. Biochimie.

[29] Maftei D., Lattanzi R., Vincenzi M., Squillace S., Fullone M.R., Miele R. (2021). The balance of concentration between Prokineticin 2β and Prokineticin 2 modulates the food intake by STAT3 signaling. BBA Adv..

[30] Yin T.C.Y., Mittal A., Buscaglia P., Li W., Sebag J.A. (2023). Activation of amygdala prokineticin receptor 2 neurons drives the anorexigenic activity of the neuropeptide PK2. J. Biol. Chem..

[31] Berruien N.N., Smith C.L. (2020). Emerging roles of melanocortin receptor accessory proteins (MRAP and MRAP2) in physiology and pathophysiology. Gene.

[32] Jackson D.S., Ramachandrappa S., Clark A.J., Chan L.F. (2015). Melanocortin receptor accessory proteins in adrenal disease and obesity. Front. Neurosci..

[33] Chaly A.L., Srisai D., Gardner E.E., Sebag J.A. (2016). The Melanocortin Receptor Accessory Protein 2 promotes food intake through inhibition of the Prokineticin Receptor-1. eLife.

[34] Fullone M.R., Maftei D., Vincenzi M., Lattanzi R., Miele R. (2022). Identification of Regions In-volved in the Physical Interaction between Melanocortin Receptor Accessory Protein 2 and Prokineticin Receptor 2. Biomolecules.

[35] Fullone M.R., Maftei D., Vincenzi M., Lattanzi R., Miele R. (2022). Arginine 125 Is an Essential Residue for the Function of MRAP2. Int. J. Mol. Sci..

[36] Rouault A.A., Lee A.A., Sebag J.A. (2017). Regions of MRAP2 required for the inhibition of orexin and prokineticin receptor signaling. Biochim. Biophys. Acta.

[37] Baron M., Maillet J., Huyvaert M., Dechaume A., Boutry R., Loiselle H., Durand E., Toussaint B., Vaillant E., Philippe J. (2019). Loss-of-function mutations in MRAP2 are pathogenic in hyperphagic obesity with hyperglycemia and hypertension. Nat. Med..

[38] Asai M., Ramachandrappa S., Joachim M., Shen Y., Zhang R., Nuthalapati N., Ramanathan V., Strochlic D.E., Ferket P., Linhart K. (2023). Loss of function of the melanocortin 2 receptor accessory protein 2 is associated with mammalian obesity. Science.

[39] Wang M., Wang X., Jiang B., Zhai Y., Zheng J., Yang L., Tai X., Li Y., Fu S., Xu J. (2022). Identification of MRAP protein family as broad-spectrum GPCR modulators. Clin. Transl. Med..

[40] Wang M., Lyu J., Zhang C. (2024). Single transmembrane GPCR modulating proteins: Neither single nor simple. Protein Cell.

[41] Hinkle P.M., Sebag J.A. (2009). Structure and function of the Melanocortin2 receptor accessory protein (MRAP). Mol. Cell. Endocrinol..

[42] Chan L.F., Webb T.R., Chung T.-T., Meimaridou R., Cooray S.N., Guasti L., Chapple J.P., Egertová M., Elphick M.R., Cheetham M.E. (2009). MRAP and MRAP2 are bidirectional regulators of the melanocortin receptor family. Proc. Natl. Acad. Sci. USA.

[43] Agulleiro M.J., Roy S., Sánchez E., Puchol S., Gallo-Payet N., Cerdá-Reverter J.M. (2010). Role of melanocortin receptor accessory proteins in the function of zebrafish melanocortin receptor type 2. Mol. Cell. Endocrinol..

[44] Sebag J.A., Zhang C., Hinkle P.M., Bradshaw A.M., Cone R.D. (2013). Developmental Control of the Melanocortin-4 Receptor by MRAP2 Proteins in Zebrafish. Science.

[45] Zhu M., Wang M., Chen Y., Zhang C. (2018). Pharmacological modulation of two melanocortin-5 receptors by MRAP2 proteins in zebrafish. J. Mol. Endocrinol..

[46] Bassi I., Luzzani F., Marelli F., Vezzoli V., Cotellessa L., Prober D.A., Persani L., Gothilf Y., Bonomi M. (2020). Prokineticin receptor 2 affects GnRH3 neuron ontogeny but not fertility in zebrafish. Sci. Rep..

[47] Chen S., Reichert S., Singh C., Oikonomou G., Rihel J., Prober D.A. (2017). Light-Dependent Regulation of Sleep and Wake States by Prokineticin 2 in Zebrafish. Neuron.

[48] Lattanzi R., Fullone M.R., De Biase A., Maftei D., Vincenzi M., Miele R. (2024). Biochemical Characterization of Prokineticin 2 binding to Prokineticin receptor 1 in Zebrafish. Neuropeptides.

[49] Chin J.W., Cropp T.A., Anderson J.C., Mukherji M., Zhang Z., Schultz P.G. (2003). An expanded eukaryotic genetic code. Science.

[50] Chen S., Schultz P.G., Brock A. (2007). An improved system for the generation and analysis of mutant proteins containing unnatural amino acids in *Saccharomyces cerevisiae*. J. Mol. Biol..

[51] Huang L., Umanah G., Hauser M., Son C., Arshava B., Naider F., Becke J.M. (2008). Unnatural amino acid replacement in a yeast G protein-coupled receptor in its native environment. Biochemistry.

[52] Meng S., Gu Q., Yang X., Lv J., Owusu I., Matrone G., Chen K., Cooke J.P., Fang L. (2018). TBX20 Regulates Angiogenesis through the PROK2-PROKR1 Pathway. Circulation.

[53] Sarfati J., Dodé C., Young J. (2010). Kallmann syndrome caused by mutations in the PROK2 and PROKR2 genes: Pathophysiology and genotype-phenotype correlations. Front. Horm. Res..

[54] Pitteloud N., Zhang C., Pignatelli D., Li J.D., Raivio T., Cole L.W., Plummer L., Jacobson-Dickman E.E., Mellon P.L., Zhou Q.Y. (2007). Loss-of-function mutation in the prokineticin 2 gene causes Kallmann syndrome and normosmic idiopathic hypogonadotropic hypogonadism. Proc. Natl. Acad. Sci. USA.

[55] Plant T.M. (2015). 60 YEARS OF NEUROENDOCRINOLOGY: The hypothalamo-pituitary-gonadal axis. J. Endocrinol..

[56] AShun-Ichiro Matsumoto S., Yamazaki C., Masumoto K., Nagano M., Naito M., Soga T., Hiyama H., Mitsuyuki M. (2006). Abnormal development of the olfactory bulb and reproductive system in mice lacking prokineticin receptor PKR2. Proc. Natl. Acad. Sci. USA.

[57] Wang M., Zhai Y., Lu L., Zhang C., Li N., Xue S., Cheng D., Fu S., Liu Q., Zhang C. (2021). Elucidation of the dimeric interplay of dual MRAP2 proteins in the zebrafish. J. Cell. Physiol..

[58] Xu Y., Li L., Zheng J., Wang M., Jiang B., Zhai Y., Lu L., Zhang C., Kuang Z., Yang X. (2021). Pharmacological modulation of the cAMP signaling of two isoforms of melano-cortin-3 receptor by melanocortin receptor accessory proteins in the tetrapod Xenopus laevis. Endocr. Connect.

[59] Li L., Xu Y., Zheng J., Kuang Z., Zhang C., Li N., Zhang C. (2021). Pharmacological modulation of dual melanocortin-4 receptor signaling by melanocortin receptor accessory proteins in the Xenopus laevis. J. Cell. Physiol..

[60] Wang M., Chen Y., Zhu M., Xu B., Guo W., Lyu Y., Zhang C. (2019). Pharmacological modulation of melanocortin-4 receptor by melanocortin receptor accessory protein 2 in Nile tilapia. Gen. Comp. Endocrinol..

[61] Tao M., Ji R.L., Huang L., Fan S.Y., Liu T., Liu S.J., Tao Y.X. (2020). Regulation of Melanocortin-4 Receptor Pharmacology by Two Isoforms of Melanocortin Receptor Accessory Protein 2 in Topmouth Culter (*Culter alburnus*). Front. Endocrinol..

[62] Wen Z.Y., Liu T., Qin C.J., Zou Y.C., Wang J., Li R., Tao Y.X. (2021). MRAP2 Interaction with Melanocortin-4 Receptor in Snake Head (*Channa argus*). Biomolecules.

[63] MacRae C.A., Peterson R.T. (2015). Zebrafish as tools for drug discovery. Nat. Rev. Drug Discov..

[64] Lee H.-C., Lin C.-Y., Tsai H.-J. (2021). Zebrafish, an In Vivo Platform to Screen Drugs and Proteins for Biomedical Use. Pharmaceuticals.

[65] Lu H., Zhou Q., He J., Jiang Z., Peng C., Tong R., Shi J. (2020). Recent advances in the development of protein–protein interactions modulators: Mechanisms and clinical trials. Signal Transduct. Target. Ther..

